# Undersampled Cine 3D tagging for rapid assessment of cardiac motion

**DOI:** 10.1186/1532-429X-14-60

**Published:** 2012-08-30

**Authors:** Christian T Stoeck, Robert Manka, Peter Boesiger, Sebastian Kozerke

**Affiliations:** 1 Institute for Biomedical Engineering, University and ETH Zurich, Gloriastrasse 35, 8092, Zurich, Switzerland; 2 Department of Cardiology, University Hospital, Zurich, Switzerland; 3 Imaging Sciences and Biomedical Engineering, King's College London, London, United Kingdom

**Keywords:** Motion analysis, Myocardial tagging, k-t undersampling, Principal component analysis, Image reconstruction

## Abstract

**Background:**

CMR allows investigating cardiac contraction, rotation and torsion non-invasively by the use of tagging sequences. Three-dimensional tagging has been proposed to cover the whole-heart but data acquisition requires three consecutive breath holds and hence demands considerable patient cooperation. In this study we have implemented and studied k-t undersampled cine 3D tagging in conjunction with k-t PCA reconstruction to potentially permit for single breath-hold acquisitions.

**Methods:**

The performance of undersampled cine 3D tagging was investigated using computer simulations and in-vivo measurements in 8 healthy subjects and 5 patients with myocardial infarction. Fully sampled data was obtained and compared to retrospectively and prospectively undersampled acquisitions. Fully sampled data was acquired in three consecutive breath holds. Prospectively undersampled data was obtained within a single breath hold. Based on harmonic phase (HARP) analysis, circumferential shortening, rotation and torsion were compared between fully sampled and undersampled data using Bland-Altman and linear regression analysis.

**Results:**

In computer simulations, the error for circumferential shortening was 2.8 ± 2.3% and 2.7 ± 2.1% for undersampling rates of R = 3 and 4 respectively. Errors in ventricular rotation were 2.5 ± 1.9% and 3.0 ± 2.2% for R = 3 and 4. Comparison of results from fully sampled in-vivo data acquired with prospectively undersampled acquisitions showed a mean difference in circumferential shortening of −0.14 ± 5.18% and 0.71 ± 6.16% for R = 3 and 4. The mean differences in rotation were 0.44 ± 1.8° and 0.73 ± 1.67° for R = 3 and 4, respectively. In patients peak, circumferential shortening was significantly reduced (p < 0.002 for all patients) in regions with late gadolinium enhancement.

**Conclusion:**

Undersampled cine 3D tagging enables significant reduction in scan time of whole-heart tagging and facilitates quantification of shortening, rotation and torsion of the left ventricle without adding significant errors compared to previous 3D tagging approaches.

## Background

Patients with myocardial infarction (MI), coronary artery disease (CAD) or impaired signal conduction in the heart such as left bundle branch block (LBBB) suffer from reduced cardiac function. In patients undergoing cardiac resynchronization therapy (CRT) [1] it is of great interest to generate a mechanical map indicating local dyssynchrony [2]. Among the various mechanical parameters, cardiac strain and torsion have been shown valuable clinical parameters in patients with MI [3] or aortic stenosis [4,5]. Cardiovascular magnetic resonance (CMR) offers a functional imaging modality to assess cardiac motion pattern and synchrony of contraction [6,7] non-invasively.

Up to date multiple approaches have been used to investigate myocardial motion such as displacement encoding with stimulated echo (DENSE) [8,9], velocity encoding [10-12], tagging by spatial modulation of magnetization (SPAMM) [13] and complementary spatial modulation of magnetization (CSPAMM) [14]. These methods can either be applied in conjunction with two dimensional (2D) or three dimensional (3D) imaging. In 2D acquisitions, multiple slices are imaged along the left ventricle (LV) and strain maps are calculated slice-by-slice. Two-dimensional acquisitions require additional techniques in order to compensate for through-plane motion. To this end, slice following [15,16], acquisition of additional orthogonal slices [17] or the encoding of through-plane displacement [18,19] (zHARP) have been proposed. While reconstruction of 3D strain patterns from two bi-planar acquisitions does require interpolation, slice-following techniques provide only a projection of true 3D motion onto a 2D subspace. Using zHARP additional gradients in through-slice direction are applied to estimate through-plane displacement by solving a set of linear equations [18].

To circumvent the need for interpolation and slice-following techniques, true 3D tagging has been proposed [20]. By applying modulation of magnetization in all three spatial directions, through-plane motion is intrinsically captured and hence no slice-following is needed. Furthermore, 3D acquisition provides full LV coverage and yields an intrinsically higher signal-to-noise (SNR) compared to 2D imaging. While cine 2D acquisitions easily fit into a single breath hold, 3D acquisitions require multiple breath holds with long duration [20]. Further approaches attempted to shorten scan time to four breath holds of 21 R-R intervals each [21] by only sampling around the harmonic peaks in k-space. These efforts, however, led to a lower spatial resolution in the two phase encoding directions. A different approach was proposed by Zhong et al. [22] using a 3D DENSE acquisition covering the entire left ventricle during free breathing. However, acquisition durations of up to 20 min [23] depending on navigator efficiency were reported and hence feasibility for routine clinical use may be questioned. Rutz et al. [24] introduced an accelerated 3D CSPAMM method only requiring three navigator gated breath holds of 18 R-R intervals each. This implementation was tested in a clinical study with patients suffering from MI and LBBB [25] providing 3D maps of synchrony and magnitude of contraction. To address issues of different breath hold levels the data obtained from respiratory navigators was used to correct for potential offsets. In their study the respiratory navigator was placed onto the right hemi-diaphragm and thereby respiratory induced displacement of the heart was approximated according to a linear relationship between liver and heart displacement [26]. The accuracy of the linear translation from motion of the lung liver interface to the position of the heart is still being debated. Nehrke et al. [27] reported a strong correlation of the displacement of the right hemi-diaphragm and the heart, but also found significant subject dependent variability in the correction coefficients especially comparing inspiration and expiration. Subject dependency of the translation of breathing induced liver motion to bulk motion of the heart has been confirmed by Moghari et al. [28].

In general, data acquisition can be accelerated by undersampling in spatial and temporal dimensions. Among the various approaches, two strategies have gained significant attention. Compressed sensing (CS) [29] employs non-linear reconstruction methods to recover information from randomly or pseudo-randomly undersampled data. Inherently, compressed sensing algorithms require incoherent sampling and hence become applicable if a sufficient number of phase-encodes exists. In case of one-dimensional tagging preparation, the number of Cartesian phase-encodes orthogonal to the tag direction can be greatly reduced providing an efficient and simple way for scan time reduction. In consequence the degrees of freedom to generate random sampling patterns become very limited and the application of compressed sensing appears less favourable in this particular application.

The second acceleration strategy involves uniform spatiotemporal undersampling in conjunction with linear reconstruction algorithms. In k-t BLAST and k-t SENSE [30] low spatial but full temporal resolution training data is used to unfold signal aliasing resulting from data undersampling. The drawback of these methods relates to temporal filtering if undersampling rates increase. To address this issue, Principal Component Analysis (PCA) of the spatial-temporal frequency domain data was introduced and results obtained with k-t PCA show improved temporal fidelity [31].

Although k-t undersampling has been extensively applied in CMR including cine and real-time imaging [32,33], perfusion [34-39] and phase contrast imaging [40-42], only few attempts of applying k-t undersampling to tagging have been reported [43,44].

The objective of the present study was to implement and test k-t undersampled whole-heart 3D CSPAMM tagging for rapid assessment of cardiac motion. The performance is demonstrated on simulated data, data obtained in healthy subjects and in patients with myocardial infarction.

## Methods

### k-t PCA

To reconstruct undersampled 3D tagging data, k-t PCA [31] is used. Similar to k-t BLAST [30], acquired data is divided into 1) training data with low spatial resolution (in phase encoding directions k_y_ / k_z_) but full temporal resolution and 2) k-t undersampled data with high spatial resolution and full temporal resolution. The training data *p*_*train*_*(x,t)* is Fourier transformed to be represented in the spatial-temporal frequency domain (*x-f*). Using principal component analysis *p*_*train*_*(x,f)* is then decomposed into a basis of temporally dependent functions *b(f*_*j*_*)* corresponding to the principal components (pc) and spatially dependent weighting coefficients *w*_*train*_*(x)* in x-pc space according to:

(1) $$p_{\mathrm{train}}\left(x_{i},f_{j}\right)=\vec{b}\left(f_{j}\right){\vec{w}}_{\mathrm{train}}{\left(x_{i}\right)}^{T}$$

The aliased signal at point (x, f_j_) resulting from R-fold undersampling can be written as:

(2) $$p_{\mathrm{alias}}\left(x,f_{j}\right)=\sum_{i=1}^{R}\vec{b}\left(f_{j}\right)\vec{w}{\left(x_{i}\right)}^{T}$$

with *w(x*_*i*_*)* denoting the spatial weighting coefficients of the unaliased image. Hence the aliased signals *P*_*alias*_*(x,f)* can be expressed as:

(3) $$P_{\mathrm{alias}}=\left[\begin{array}{c} p_{\mathrm{alias}}\left(x,f_{1}\right)\\ p_{\mathrm{alias}}\left(x,f_{2}\right)\\ \ldots \\ p_{\mathrm{alias}}\left(x,f_{N}\right) \end{array}\right]=E{\vec{w}}_{x}\text{with}{\vec{w}}_{x}=\left[\begin{array}{c} {\vec{w}}^{T}\left(x_{1}\right)\\ {\vec{w}}^{T}\left(x_{2}\right)\\ \ldots \\ {\vec{w}}^{T}\left(x_{R}\right) \end{array}\right]\text{and}E=\left[\begin{array}{c} \vec{b}\left(f_{1}\right)\\ \vec{b}\left(f_{2}\right)\\ \ldots \\ \vec{b}\left(f_{N}\right) \end{array}\right]$$

Finally, the unaliased spatial weighting coefficients are obtained by solving:

(4) $$w_{x}=\Theta E^{H}{\left(E\Theta E^{H}+\psi \right)}^{+}P_{\mathrm{alias}}$$

where *Θ* represents an estimate of *w*_*x*_ from training data, *Ψ* denotes noise variance, *H* conjugate transpose and + the Moore-Penrose pseudo-inverse.

### Computer simulation

**Figure 1:**
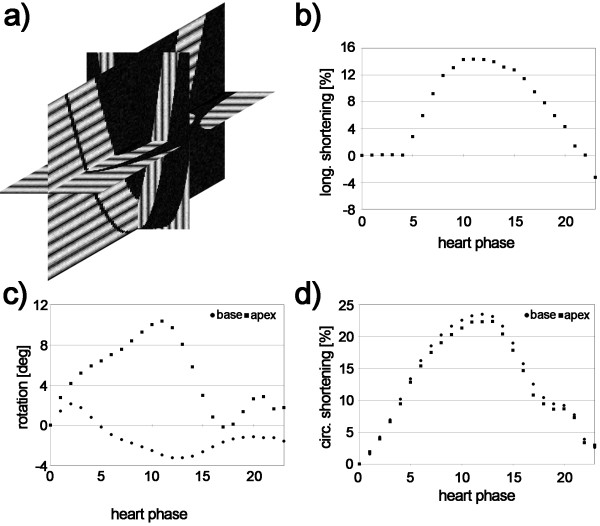
**Numerical simulation.** Three stacks with orthogonal line tag pattern were simulated (**a**). As input for the model, longitudinal shortening (**b**), rotation (**c**) and circumferential shortening (**d**) obtained from one in-vivo acquisition were used.

**Figure 2:**
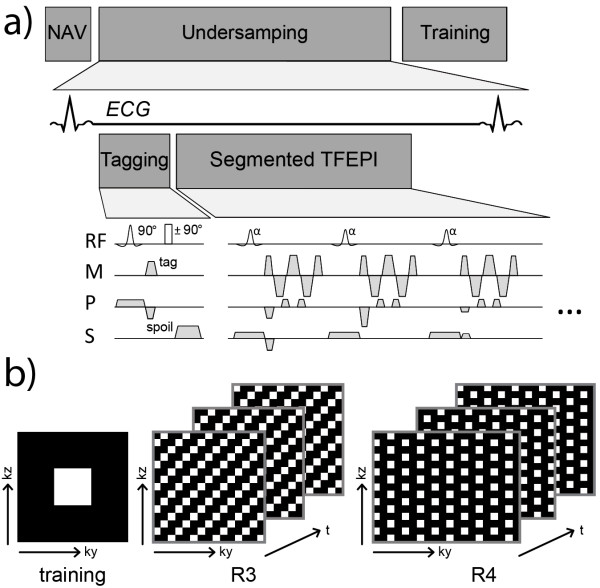
**Sequence diagram (a) and sampling pattern for k-t undersampling (b).** After detection of the R-wave the tagging preparation is applied, followed by two repetitions of undersampled data and training data acquisitions for each stack.

All simulations were performed in Matlab (The MathWorks, Natick, MA, USA). Three orthogonal stacks with line tagging modulation in readout direction were generated (Figure 1a). The CSPAMM method [14] was simulated to avoid tag line fading. The model consisted of a contracting left ventricle as well as static tissue representing chest wall and liver. Circumferential shortening and rotation as measured in a healthy subject at basal and apical level was linearly interpolated along the long-axis to create three-dimensional motion data. Peak circumferential shortening was 18.8% and 17.8% for base and apex, respectively. Peak rotation was −3.2° and 10.3° for base and apex (Figure 1b-d). Longitudinal shortening obtained from the same in-vivo subject was incorporated. Simulations of undersampled data acquisition were compared to fully sampled simulated data sets with equivalent spatial and temporal resolution. The matrix size was set according to practical values [24,25] (Table 1). Gaussian noise was added to k-space data before undersampling resulting in a SNR of 25 prior to undersampling. Both undersampled and training data were extracted from the computer model (Figure 2b). Undersampling rates of R = 3, 4, 5 and 8 were simulated. In all simulations, five training profiles were used in k_y_ and k_z_ direction resulting in a total of 25 training profiles.

**Table 1:** Parameters used for numerical simulation

**Undersampling factor**	**Matrix size k**_**x**_ **× k**_**y**_	**Slices k**_**z**_	**Heart phases**	**Training profiles (k**_**y**_ **× k**_**z**_**)**	**R**_**net**_
3	65 × 15	15	24	25 (5 × 5)	2.3
4	65 × 20	15	24	25 (5 × 5)	3
5	65 × 20	15	24	25 (5 × 5)	3.5
8	65 × 16	15	24	25 (5 × 5)	4.4

**Figure 3:**
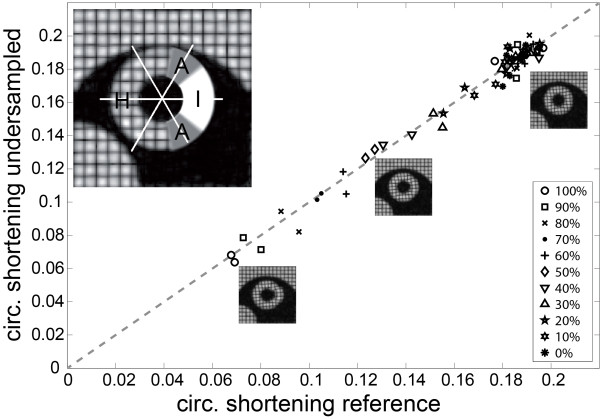
**Comparison of peak circumferential shortening from undersampled (R = 3) and fully sampled simulated data.** Myocardium was divided into three groups: infarction (I), which covers a sector of 80° (22% of myocardium), healthy tissue (H) and infarct adjacent (A) tissue “attaching” infarcted to healthy tissue over a sector of 40° (11% of myocardium). For infarcted tissue, different degrees of immobilized tissue ranging from 0% to 100% were simulated. The dashed line indicates the reference diagonal with slope 1.

In order to study regional wall motion abnormalities, myocardial infarctions of different severity were simulated. To this end, radial shortening in the lateral sector was changed from 100% (no infarction) to 0% (completely static) in steps of 10% (Figure 3). The reduction of myocardial motion in the infarcted zone was applied transmurally along the entire long axis of the left ventricle. Reduction of radial shortening directly reduced the circumferential contraction. In order to assure a smooth transition between infarcted and healthy tissue, the infarcted tissue was continuously “attached” to the adjacent healthy tissue, by reducing the motion damping factor continuously over a sector of 40° on both ends of the infarcted region.

### In-vivo measurements

Eight healthy subjects (7 male, age: 27.5 ± 3.5 years) and five patients with myocardial infarction (5 male, age: 54.8 ± 5.9 years, 1 patient with acute myocardial infarction post percutaneous coronary intervention (PCI), 4 patients with chronic myocardial infarction) were studied. Imaging was performed on a 1.5 T Philips Achieva System (Philips Healthcare, Best, the Netherlands) using a 5 channel cardiac receiver array. Written informed consent was obtained from all subjects and the protocol was approved by the institutional review and ethics boards.

Tagged images were acquired using an ECG triggered multi-shot EPI sequence with sequence parameters listed in Table 2. As illustrated in the sequence diagram in Figure 2 the tagging preparation was applied within 10 ms after the detection of the R-wave. As the first RF pulse used for tagging preparation was applied selectively in phase encoding direction, signal outside the field-of-view was suppressed after CSPAMM subtraction and hence reduced field-of-view acquisition could be performed [16]. The imaging sequence was repeated to acquire three orthogonally tagged stacks. For each stack 21–24 heart phases were recorded.

**Table 2:** Imaging parameters for the acquisition of fully sampled reference and undersampled data (R = 3 and 4)

**Undersampling factor**	**Fully sampled / R3**	**Fully sampled / R4**
Matrix size k_x_ × k_y_	28 × 15	28 × 20
Phase-encodes k_z_	15	15
Receiver bandwidth	314 Hz/pixel	314 Hz/pixel
Spatial resolution	3.8 × 7.2 × 7.2 mm^3^	3.8 × 5.4 × 7.2 mm^3^
Heart phases	21	24
Temporal resolution	30.3 ms	30.3 ms
EPI factor	5	5
TFE factor	5	5
TE/TR	2.8 ms/6.1 ms	2.8 ms/6.1 ms
R-R intervals_undersampled_	18	18
R_net_	2.25	3
Training profiles (k_y_,k_z_)	25 (5/5)	25 (5/5)
R-R intervals_training_	6	6
R-R intervals_full_	3x18	3x18
Tag line distance	7 mm	7 mm

Fully sampled data was acquired in three consecutive breath holds. To guarantee similar breath hold position for each tagged stack a pencil beam respiratory navigator placed on the right hemi-diaphragm was used. The position of the lung liver interface was monitored at the beginning of each breath hold and displacements within a 5 mm gating window were accepted. The breath hold was repeated if the subject’s breath hold level was not within the given window. Retrospective undersampling was applied with reduction factors of R = 3 and 4. Prospectively undersampled data were acquired according to the schematic shown in Figure 2b in an additional breath hold. To facilitate comparison, all imaging parameters except for undersampling factors were kept identical for fully sampled and undersampled data acquisitions. For undersampling factors R = 3 and 4 the corresponding sets of parameters are given in Table 2. Prior to image reconstruction, the 5-channel coil array data were compressed into a single virtual coil data set using the array compression method proposed by Buehrer et al. [45].

### Data analysis

Epicardial and endocardial contours were manually drawn for each slice of interest and a midmural contour was calculated. This contour was tracked over time using the peak combination harmonic phase (HARP) [46,47] algorithm implemented in an in-house software. Rotational motion and circumferential shortening was calculated from tracked contours as previously described by Ryf et al. [48]. Curves of circumferential shortening and rotation over the cardiac cycle were fitted by 8^th^ order polynomials, and maxima were found by estimating roots of the derivative. From the mean rotation over the contour, torsion was derived by taking the difference in peak rotation at the most apical and the most basal level [49]. Analysis of peak circumferential shortening and rotation was performed in six sectors per slice in 9 slices for computer simulation and 8–9 slices in in-vivo experiments. Comparisons were performed sector-wise and are reported for the entire LV.

For simulation the tracking results from fully sampled data were used as ground truth reference. For in-vivo measurements the mean of paired data points was used as reference. In order to facilitate comparison the initial contour was kept the same for the fully sampled reference and the retrospectively undersampled data in simulation and in-vivo. In the comparison of fully sampled data with prospectively undersampled acquisitions, contours were redrawn to account for changes in breath hold position and/or patient motion in-between scans.

Analysis of simulated data was done by calculating the relative difference defined as: $error_{\mathrm{rel}}=\left|max\left(motion_{R=1}\right)-max\left(motion_{R>1}\right)\right|/\left|max\left(motion_{R=1}\right)-min\left(motion_{R=1}\right)\right|$. The fully sampled data was used as reference. Relative differences ± one standard deviation are reported in % for circumferential shortening and rotation. In order to estimate the correlation between undersampled and reference data linear regression was performed correlating circumferential shortening and rotation of reference data and undersampled data. Regression slope, offset and the corresponding 95% confidence interval were estimated as well as the correlation coefficient R^2^ and the standard error of the estimate (SEE). The SEE is given in % of the range of motion and is defined as $SEE=\sqrt{\frac{1}{N-2}\sum_{i=1}^{N}{\left(x_{i}-{x^{\prime }}_{i}\right)}^{2}}$ with *N* being the number of points, *x*_*i*_ denoting measurement data and *x*_*i*_*’* corresponding values derived from linear regression. For in-vivo imaging, peak circumferential shortening and rotation were compared using Bland Altman analysis. Mean differences and the 95% levels of agreement corresponding to 2 standard deviations (2SD) are reported. Linear regression was performed on peak circumferential shortening and rotation. Regression slopes, offsets, correlation coefficients and the SEE are reported.

Comparing reference data and retrospectively undersampled data, the HARP tracking performance of contour coordinates was studied based on contour vertex definitions in polar coordinates. Similar to the analysis of peak circumferential shortening and rotation, Bland Altman and linear regression analysis were performed.

Patient data are reported using bull’s-eye plots of peak circumferential shortening, and late gadolinium enhancement (LGE). The centre of the bull’s-eye plots represents the apex and the outer ring the base of the LV. For LGE images the average myocardial signal intensity per slice and sector normalized to the signal intensity measured in infarcted tissue is shown. Sectors were grouped into two groups: 1) non-viable sectors having more than 50% of the area presenting LGE and 2) viable sectors having less or equal to 50% of the area presenting LGE. Peak circumferential shortening was estimated for all sectors in each group and the median, 50^th^ percentiles and 90^th^ percentiles were estimated and presented in box-plots. Statistical significance of the differences in peak circumferential shortening between both groups were estimated by a two-tailed Wilcoxon signed-rank test. The results were Bonferroni corrected for repeated testing. A p-value less than 0.05 was considered statistically significant.

## Results

### Computer simulation

**Figure 4:**
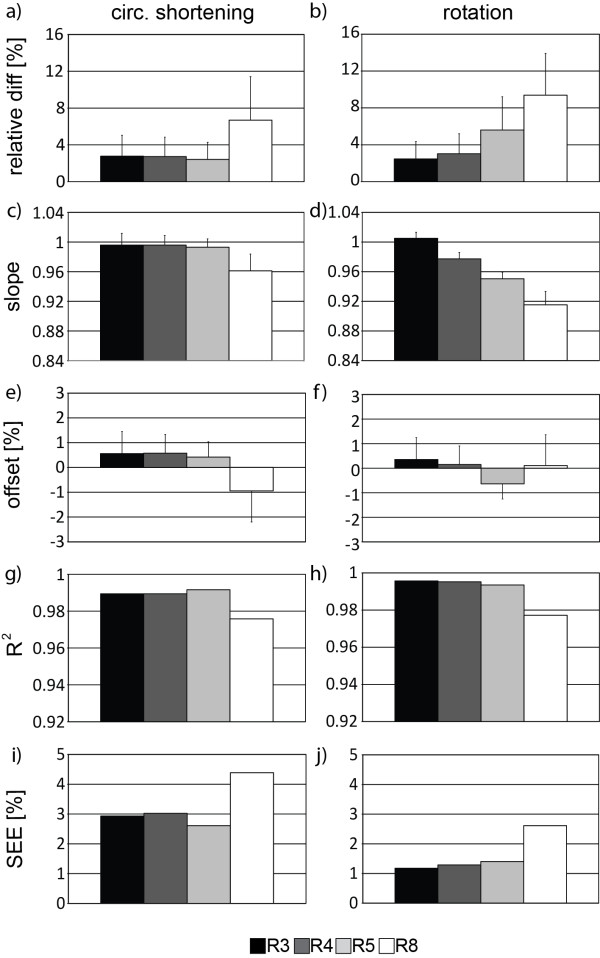
**Comparison of circumferential shortening and rotation for fully sampled reference data and retrospectively undersampled data from computer simulation.** Relative differences are presented as average over the entire LV (**a-b**) along with slope and offset of linear regression and the corresponding correlation coefficient R^2^ and the standard error of the estimate (SEE) (**c-j**).

Normalized differences in peak circumferential shortening and rotation between undersampling factors of R = 3, 4, 5 and 8 and fully sampled data used as reference are shown in Figure 4a-b. Errors in circumferential shortening and rotation were below 5% for undersampling factors of R = 3–5 and R = 3, 4, respectively. At R = 8 errors in circumferential shortening and rotation were 2.5 and 3.1 times greater compared to data obtained with R = 4. The differences in torsion between undersampled and fully sampled data were −0.13°, 0.42°, 0.95° and 1.85° for R = 3, 4, 5 and 8, respectively.

Figure 4c-j shows regression slope, offset, correlation coefficient and SEE for R = 3, 4, 5 and 8. The 95% confidence intervals are presented as error bars for fitted slopes and offsets.

Figure 3 shows the result of motion tracking in the presence of simulated infarction. Peak circumferential shortening in an equatorial slice is plotted for the reference vs. undersampled data. Linear regression was performed, resulting in a slope of 1.01 (±0.04 95% confidence interval) and an offset of −2.01% of the range of contraction (±4.79% 95% confidence interval).

### In-vivo measurements

**Figure 5:**
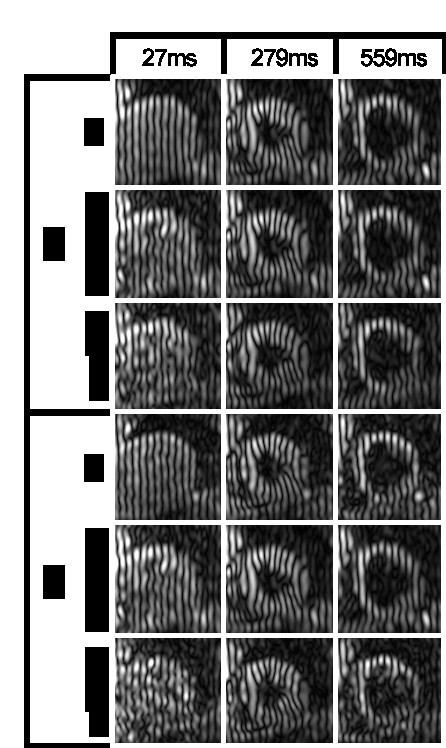
**Comparison of short-axis views reconstructed from fully sampled (ref) and 3- and 4-fold retrospectively (retrospect) and prospectively (prospect) undersampled data.** Three different time points are shown.

Figure 5 shows short axis slices reconstructed from fully sampled and 3- and 4-fold retrospectively and prospectively undersampled data. Frames at 27 ms after detection of the R-wave, at end systole (279 ms) and at mid diastole (559 ms) are shown.

**Figure 6:**
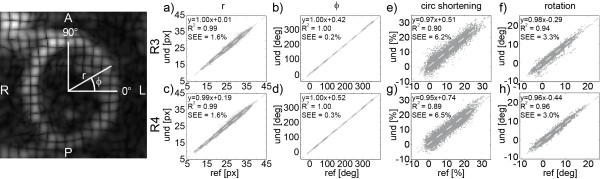
**Linear regression analysis for point-wise and sector-wise comparison of fully sampled (ref) and retrospectively undersampled (und) data in healthy volunteers (R = 3 and 4).** Point-wise comparison was performed for polar coordinates r (**a, c**) and ϕ (**b, d**). For circumferential shortening (**e, g**) and rotation (**f, h**) entire motion curves were compared per sector across the entire LV.

Linear regression analysis performed on fully sampled and retrospectively undersampled data is shown in Figure 6. For each regression the equations of the linear fit, the correlation coefficients R^2^ and the SEE are given. Figure 6a-d show the analysis of polar coordinates of tracked points and Figure 6e-h demonstrate sector-wise comparison of time curves of circumferential shortening and rotation for R = 3 and 4.

**Figure 7:**
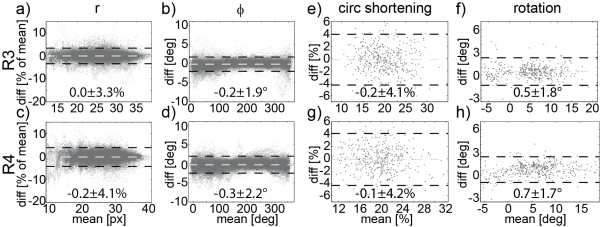
**Bland Altman plots of point-wise and sector-wise comparison of fully sampled and retrospectively undersampled in-vivo data (R = 3 and 4) in healthy volunteers.** Mean differences (grey) and 95% levels of agreement (2SD) (black) are indicated by the dashed lines. Analysis was performed on the entire LV. Point-wise comparison was performed for the polar coordinates r (**a, c**) and ϕ (**b, d**) for each tracked point. Circumferential shortening (**e, g**) and rotation (**f, h**) were compared per sector.

In Figure 7 shows the Bland-Altman comparison of fully sampled and retrospectively undersampled in-vivo data for R = 3 and 4. Dashed lines represent the mean difference (light grey) and the 95% limit of agreement (2SD) (black). In Figure 7a-d provides the comparison of radial coordinates (r = radius and ϕ = angle) of tracked contour points. Mean differences ± 2SD of r were 0.0 ± 3.3% and −0.2 ± 4.1% for R = 3 and 4 and −0.2 ± 1.9° and −0.3 ± 2.2° for ϕ. In Figure 7e-h Bland-Altman plots for peak circumferential shortening and peak rotation are given. The mean differences ± 2SD for circumferential shortening were −0.2 ± 4.1% and −0.1 ± 4.2% for R = 3 and 4. For rotation, mean differences ± 2SD were 0.5 ± 1.8° and 0.7 ± 1.7° for R = 3 and 4. The mean differences of torsion were 0.45 ± 2.22° (p = N.S.) and 0.05 ± 2.24° (p = N.S.) for R = 3 and 4, respectively.

**Figure 8:**
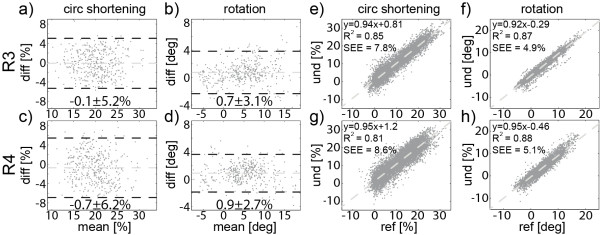
**In-vivo results of the comparison between fully sampled reference data (ref) and data acquired with an acceleration rate of R = 3 and 4 (und).** Mean differences (grey) and 95% level of agreement (2SD) (black) are shown for peak circumferential shortening (**a, c**) and rotation (**b, d**) of the entire left ventricle. Motion curves of circumferential shortening (**e, g**) and rotation (**f, h**) of undersampled and reference data are plotted against each other and linear regression was performed.

Figure 8 shows the resulting Bland-Altman analysis of peak motion (a-d) and correlation between full time curves of motion (e-h) comparing fully sampled data with data acquired with prospective undersampling (R = 3 and 4). Mean differences ± 2SD of peak circumferential shortening were −0.1 ± 5.2% and −0.7 ± 6.2% for R = 3 and 4. Mean differences of peak rotation were found to be 0.44 ± 1.80° and 0.73 ± 1.67° for R = 3 and 4 while differences in torsion were 0.48 ± 4.20° (p = N.S.) and 0.03 ± 4.48° (p = N.S.) for R = 3 and 4, respectively.

**Figure 9:**
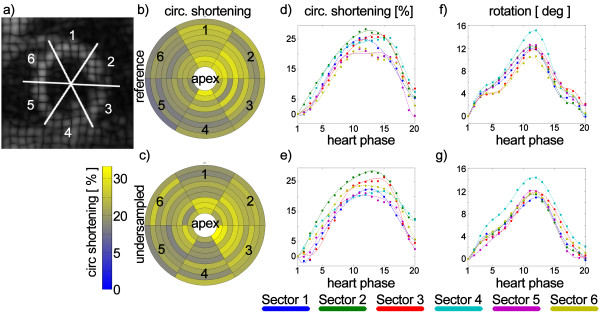
**Comparison of circumferential shortening for fully sampled data and 4-fold undersampled data.** The first column shows maps of peak circumferential shortening for fully sampled **b**) and undersampled data **c**). Resulting motion profiles taken from an equatorial slice are shown for circumferential shortening (**d**, **e**) and rotation (**f**, **g**). The dots represent the actual data points while the line represents the polynomial fit.

Bull’s-eye plots of peak circumferential shortening and profiles of circumferential shortening and rotation derived from undersampled (R = 4) and fully sampled data are compared in Figure 9 for one healthy subject. Spatially depend differences are not observed.

**Figure 10:**
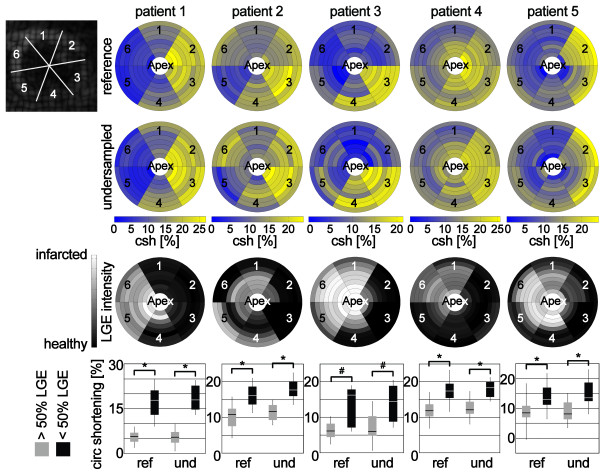
**Maps of peak circumferential shortening (csh) for five patients with myocardial infarction.** The top row represents results from fully sampled data while the second row shows data from prospectively undersampled acquisitions. The third row shows the result of late gadolinium enhancement imaging (LGE). The myocardial signal intensity post contrast was averaged over each sector and normalized to the signal intensity from completely infarcted tissue. The bottom row shows box-plots of peak circumferential shortening for sectors with more than 50% late gadolinium enhanced signal (grey) and sectors with less than 50% late gadolinium enhanced signal (black). The box represents 50% of the data points and the error bars 90%. Statistical significance is indicated by # for p < 0.002 and * for p < 0.0002.

Figure 10 compares circumferential shortening obtained from undersampled and fully sampled reference data for the five patients in relation the LGE findings. Differences in peak circumferential shortening in sectors with more than 50% of their area presenting LGE and sectors with less than 50% LGE were statistically significant in all patients. Mean difference ± 2SD in torsion between undersampled and fully sampled reference data in patients was 0.72 ± 2.14°. The SNR in the fully sampled in-vivo data was 36 ± 12 on average.

## Discussion

In this work undersampled 3D CSPAMM tagging in combination with k-t PCA has been implemented and validated.

The analysis of simulation results has revealed a maximum applicable acceleration factor of four. Generally, good correlation was found between fully sampled and retrospectively undersampled data over the entire left ventricle. Analysis of relative differences in circumferential shortening showed a slight decrease in error going from the apex towards the base of the heart. As motion was normalized for each slice, this observation is associated with the low magnitude of contraction of apical myocardium.

Different transmural extensions of infarcted tissue were investigated by computer simulation. Motion analysis showed a good correlation between undersampled and fully sampled reference data. Transmural reduction of contraction as low as 20% could be distinguished from healthy fully contracting tissue. Although fully infarcted tissue was simulated as well, sector-wise analysis always showed contraction greater than 6%. This is due to the choice of the position and size of the sectors used for analysis. Sectors with non-contracting tissue contained slightly contracting neighbouring tissue, which leads to a non-zero average circumferential shortening within a sector.

In vivo comparison between fully sampled data and retrospectively undersampled data showed good agreement as the mean difference was less than 1% (% of range of motion). Differences between reference data and prospectively undersampled acquisition were found to be larger in comparison to differences seen relative to retrospectively undersampled data. This finding is related to multiple issues. On one hand, reference data and undersampled data were acquired in two consecutive scans. Despite the use of a gating respiratory navigator, different breath hold levels are possible and slices used for analysis might hence differ in position. On the other hand the fully sampled reference data itself was acquired in three consecutive breath holds, which, despite the use of a respiratory gating window of 5 mm, can lead to stack misalignment within the reference data. The resulting circumferential shortening is dependent on the transmural position of the tracked contour. If stack misalignment occurs a contour that appears to be midmural in one stack can be positioned more epicardially or endocardially in the consecutive two stacks from which the motion orthogonal to the first stack is derived from. Hence a contour point is tracked at three different transmural positions. Therefore the motion profiles obtained from fully sampled data, used as gold standard reference in this work, might have been compromised. Image registration was not performed in this study. The implementation of tailored registration algorithms that can handle orthogonal tagging modulations requires further investigation.

Maps of peak circumferential shortening as well as the corresponding motion profiles were found to agree well for reference and undersampled data. The magnitude of circumferential shortening is comparable to previously reported values [24,25,50,51]. Besides potential motion smoothing, expressed by the positive mean difference for rotation, a slice by slice comparison of prospectively undersampled and fully sampled data might have been biased by an offset in breath hold level for both acquisitions.

In this study, standard 2D single slice tagging data were not available for reference. In order to capture through-plane motion and hence make data comparable to 3D tagging, non-standard extensions of 2D tagging such as slice following [15] or zHARP [18] are required. Accordingly, assessment of error in the present work was relative to fully sampled 3D CSPAMM data only, which is a clear limitation. Nevertheless, there have been previous records of validating 3D CSPAMM on healthy subjects and patients, which may serve as benchmark [24,25].

Spatial resolution in each of the three orthogonal stacks in 3D CSPAMM may be considered coarse (3.8 × 5.4-7.2 × 7.2 mm^3^). The resolution given in readout direction is, however, directly linked to the tagline spacing when using HARP analysis. Higher temporal resolution (< 30 ms) is desirable as this allows separating data in x-pc space further and hence improves reconstruction accuracy in k-t PCA. However, this results in more heart phases and hence more RF excitations per cardiac cycle reducing the contrast-to-noise ratio of the tagged data.

Five patients with myocardial infarction were examined using undersampled tagging and LGE imaging. Maps of circumferential shortening derived from reference data and prospectively undersampled data agreed well and infarcted regions could be localized. Direct correlation of peak circumferential shortening with the area of delayed enhancement was found to be only moderate. A potential limitation of infarct detection is related to averaging of motion within sectors. A six-sector per slice model [24,25] results in 16.7% of total myocardial mass captured per sector and per slice. For example, non-viable tissue in border zones of infarction is passively moved and compressed and hence the extent of motion abnormality may be overestimated. This issue may be addressed by increasing the number of sectors per slice and by using multiple circumferential contours covering the entire transmural extent in future work.

Several strategies may be envisioned to shorten the relatively long breath hold durations (~20 sec) of the present protocols. First, sampling of training data can be fully integrated into the acquisition of undersampled data. Such a variable density EPI approach provides shorter overall scan duration at the expense of reduced temporal resolution. Second, the separation of training data and undersampled data allows splitting the data acquisition into two breath holds. Compared to the method proposed by Rutz et al. [24] the three stacks are not acquired within three consecutive breath holds, but could be acquired within one breath hold for high resolution undersampled and a second breath hold for low resolution training data. Since the training data has very low spatial resolution in the phase-encode directions (2.3 × 2.3 cm^2^), differences in breath hold levels between acquisitions no longer pose a concern.

## Conclusion

Undersampled cine 3D tagging in conjunction with k-t PCA reconstruction enables significant reduction in scan time of whole-heart tagging and facilitates efficient and accurate quantification of shortening, rotation and torsion of the left ventricle. Using 3-fold undersampling the entire cine 3D tagging acquisition could be accommodated in a single breath hold and feasibility in volunteers and patients was demonstrated. Future work is dedicated to shorten breath hold durations further and to apply the method in larger patient cohorts to prove clinical value.

## Abbreviations

CMR: Cardiovascular magnetic resonance; MI, Myocardial infarction; CAD, Coronary artery disease; LBBB, Left bundle branch block; CRT, Cardiac resynchronization therapy; LV, Left ventricle; PCI, Percutaneous coronary intervention; LGE, Late-gadolinium enhancement; DENSE, Displacement encoding; SPAMM, Spatial modulation of magnetization; CSPAMM, Complementary spatial modulation of magnetization; HARP, Harmonic phase; PCA, Principal component analysis; SNR, Signal to noise ratio; SEE, Standard error of the estimate; ref, Reference; und, Undersampling; csh, Circumferential shortening.

## Competing interests

The authors declare that they have no competing interest.

## Authors’ contributions

CS: study design, data acquisition, reconstruction, image analysis, manuscript drafting, statistics, and literature research. RM: study design, image analysis, statistics and revision of manuscript. PB: study design, literature research, and revision of manuscript. SK: Study design, reconstruction, literature research and revision of manuscript. All authors read and approved the final manuscript.

## Acknowledgments

The authors acknowledge funding by the Swiss National Science Foundation, grant #CR3213_132671/1.
